# Effects of a Lifestyle Intervention on Health-Promoting Behavior, Psychological Distress and Reproductive Health of Overweight and Obese Female College Students

**DOI:** 10.3390/healthcare9030309

**Published:** 2021-03-10

**Authors:** Ju-Hee Nho, Soo-Wan Chae

**Affiliations:** 1 College of Nursing, Jeonbuk National University, Jeonju 54896, Korea; 2 Clinical Trial Center for Functional Foods, Jeonbuk National University Hospital, Jeonju 54907, Korea; swchae@jbctc.org

**Keywords:** overweight, obesity, female, program, lifestyle

## Abstract

This study aimed to examine the effect of a lifestyle intervention (LSI) on overweight or obese female university students. Participants: A total of 53 overweight or obese female college students participated. This study was conducted from May to December 2017 in Jeonbuk Province, South Korea. A quasi-experimental design using a non-equivalent control group pretest-posttest was used. The LSI consisted of providing health information, individual health counseling, lifestyle monitoring, and effective support based on the interaction model of client health behavior, which was implemented for 12 weeks. Significant group differences were found in health-promoting behavior, psychological distress, reproductive health, body weight, body fat, and triglyceride level among participants. LSIs are effective in improving health-promoting behavior, psychological distress, reproductive health, and body composition. Therefore, healthcare providers should develop and apply LSIs through interaction for overweight or obese female college students.

## 1. Introduction

In the United States, the prevalence of obesity in women aged 20–39 was 36.5% in 2015–2016, and it has been increasing since 1999 [1]. In South Korea, the prevalence of obesity in women aged 19–29 continuously increased from 13.8% in 2016 to 16.2% in 2017 [2]. Weight gain leads to the risk of cardiovascular disease, type 2 diabetes, cancer, osteoarthritis, asthma, psychological stress, and reproductive health issues across the population; college students are not resistant to the health impact of such weight gain [3]. Overweight and obese college students, particularly women, have a 12% and 33% higher risk of experiencing blood pressure and coronary heart issues, respectively, than normal-weight individuals [4]. In addition, overweight and obese female students have higher rates of depression, stress, panic disorders, stigmatization, and lower academic achievement [5,6]. Further, obesity with women impacts reproductive health, as it is associated with an increased risk of amenorrhea, irregular menstruation, infertility, and pregnancy complications (e.g., stillbirth and miscarriage) [7,8].

College students face various challenges during this life period; they must adapt to new situations, create new environments and social networks, and develop autonomy [9]. Healthy lifestyles formed during this period have a great influence on an individual’s future health. Despite the importance of creating healthy habits as young adults, most students usually start drinking and smoking after entering college, and they adopt unhealthy lifestyles, including detrimental eating habits, lack of exercise, and ineffective stress coping mechanisms [10]. In a study of college students, only 38% of the females consumed three meals a day; and they ate more sweets and drank more wine or beer than males [11]. Women in their 20s had only 14.5% of the required muscular exercise and 9.9% of the aerobic physical activity and strength exercise; this was twice as low as that of men [12]. In addition, female students have lower health-promotion behaviors for physical activity and nutrition than male students [13]; therefore, it is necessary to make efforts to improve female students’ healthy lifestyles.

Lifestyle in female college students is related to psychological and reproductive health. In a study of 275 female students, more than 44% had depression and anxiety, which were positively correlated with healthy lifestyles [14]. In addition, healthy lifestyles were associated with high reproductive health knowledge, attitudes, and behaviors, and regular menstruation [14]. In another study of 250 female university students in Iran, there were differences in diet, physical activity, and social relationships between subjects with and without dysmenorrhea [15].

Various lifestyle interventions (LSI) for overweight and obese college students have been reported to be effective in many areas. In a review of LSIs among college students, LSIs resulted in improvement in weight loss, fruit and vegetable consumption, health behaviors, and physical fitness [16]. In the Project WebHealth study of 653 undergraduate students at 8 universities in the United States, the weight-related health behaviors of the participants improved [17]. In a randomized study of 417 university students, vitamin, fruit, egg, mild, and diary products increased while processed food intake decreased [18]. However, despite the ongoing obesity control effect, the prevalence of overweight and obesity in female college students keeps increasing, and health-related problems continue.

It has been reported that the lifestyle of female college students is related to their physical, psychological, and reproductive health. Accordingly, it is necessary to develop and implement LSI programs to improve female college students’ physical, psychological, and reproductive health.

Thus, the purpose of this study was to (a) develop an LSI and (b) identify the effects of the intervention on the following outcomes: health-promoting behavior, psychological distress, and reproductive health of overweight and obese female college students.

## 2. Materials and Methods

### 2.1. Design

The study followed a quasi-experimental design using a non-equivalent control group pretest-post design to investigate the effects of an LSI on overweight and obese university women. A research notice, including the researcher’s contact information, was posted on the online and offline bulletin boards of universities to recruit participants. Among the students who met the inclusion criteria, female students who agreed to participate in the research were included. The investigator provided information on the research (e.g., research progress, methods, period, data collection procedures, benefits and harms from the research, and compensation); after obtaining consent for research, data were collected. The students who were in contact with the investigators at university A were assigned to the experimental group, students in contact at university B were assigned to the control groups.

### 2.2. Participants

The participants were overweight and obese women from two universities in Jeonbuk, South Korea. The study took place from May to December 2017. The inclusion criteria were (i) being 20–30 years old and (ii) having a body mass index (BMI) ≥ of 23 kg/m^2^. The exclusion criteria included (i) being diagnosed with a neurological disorder (e.g., paresis, stroke, Parkinson’s disease), (ii) acute heart impairment (e.g., uncontrolled hypertension, congestive heart failure), (iii) unstable chronic illness (e.g., uncontrolled diabetes mellitus, malignancies), (iv) severe musculoskeletal impairment (e.g., inability to participate in the programs), and (v) refusal to participate in the study.

**Figure 1:**
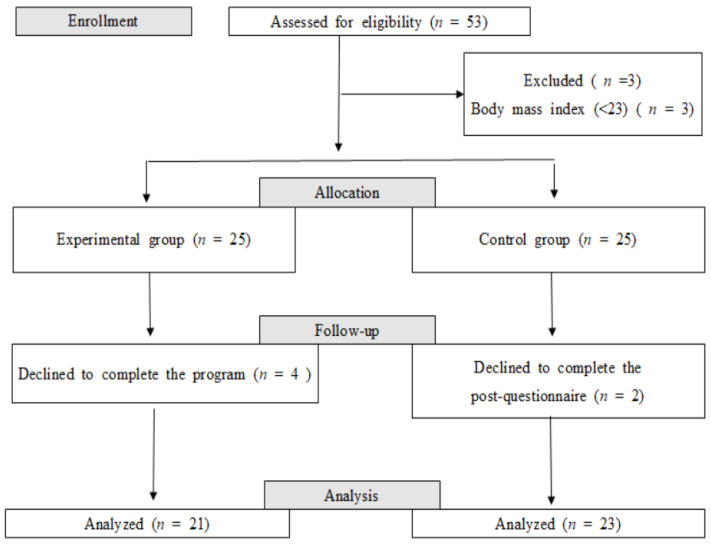
Flow diagram of participant enrollment in the study.

The G*power 3.1.9.2 statistical analysis was used to estimate the sample size for the t-test analysis with a significance level of 0.05, power of 0.8, and effect size of 0.8. The effect size of 1.0 was based on a previous LSI for obese and overweight women [19]. The minimum sample size was calculated as 21 per group. A total of 50 participants were selected, with 25 subjects in each group, to deal with a predicted dropout rate of 20%. A total of 53 women were screened, three of whom were excluded because of body mass index < 23 kg/m^2^. The final 50 women were assigned to either the experimental (n = 25) or the control (n = 25) groups. After completing the pretest, four subjects in the experimental group declined to participate in the program; in the control group, two subjects declined to complete the posttest. Finally, 44 women participated, 21 in the experimental group and 23 in the control group, with a 17.0% dropout rate (Figure 1). 

### 2.3. Conceptual Framework

**Figure 2:**
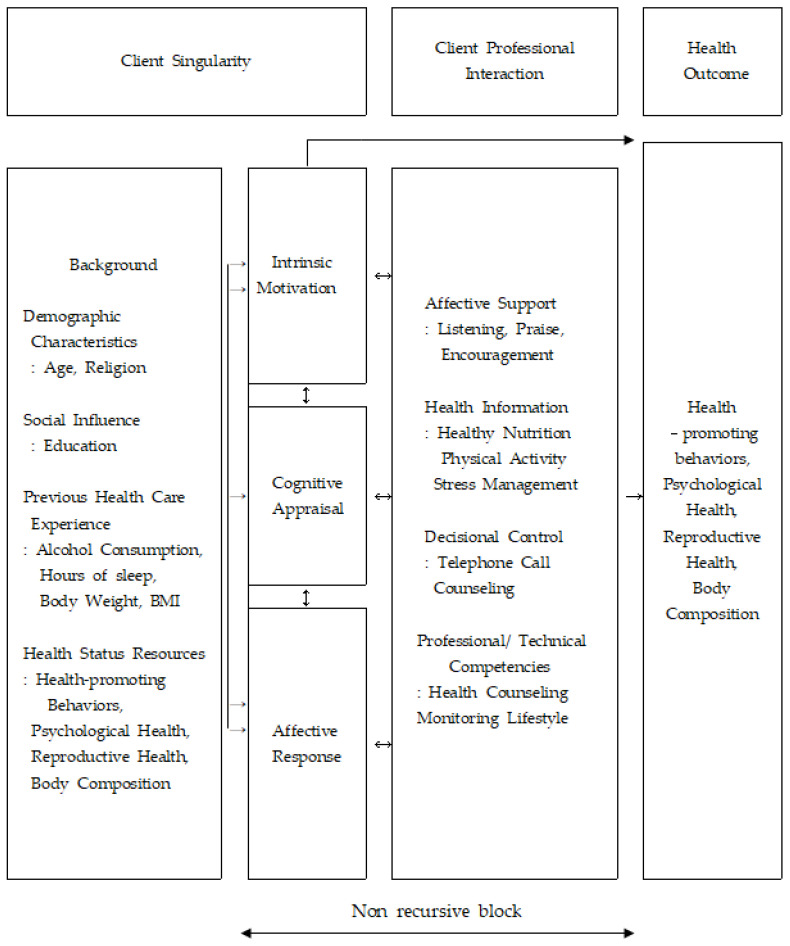
Conceptual framework.

In this study, Cox’s interaction model of client health behavior (IMCHB) [20] was applied to develop an LSI to improve health-promoting behaviors, psychological and reproductive health through systematic and continuous interaction with professionals. The IMCHB emphasizes client singularity, client professional interaction, and health outcomes [19]. The client’s singularity includes background, social influence, previous healthcare experience, environmental resources, and health status resources. Therefore, we collected information on age, religion, education, alcohol assumption, hours of sleep, body weight, BMI, and health status of women. Intrinsic motivation, cognitive appraisal, and affective response were linked to the health outcome since they would affect the health outcome by providing intervention. Affective support as an element of client-professional interaction refers to attending to the client’s level of emotional arousal, which includes listening, praise, and encouragement. The health information contained healthy nutrition, physical activity, and stress management. Decisional control refers to the individual’s expectations of having the power to make decisions based on the consequences, which included telephone call counseling. Moreover, professional/technical competencies are promoted by professionals via health counseling and monitoring of lifestyle. Health outcomes were measured for health-promoting behaviors, psychological, reproductive health, and body composition (Figure 2).

### 2.4. Lifestyle Intervention based on the Interaction Model of Client Health Behavior

We developed an LSI based on the IMCBH based on previous literature on LSI [19,21].

The LSI was composed of 12 weekly sessions, four categories of health information (nutrition, physical activity, and stress management), individual health counseling, lifestyle monitoring, and affective support [19,21].

The health information session was conducted seven times, addressing the importance of a healthy lifestyle (nutrition, physical activity, and stress management) for physical, psychological, and reproductive health by the group. Nutritional education focused on the weight control diet, diet behaviors, and diet recipes. Physical activity was conducted by registered physiotherapists on flexibility, resistance, and neurovascular training. The stress management intervention included knowing oneself and strategies for self-respect. Further, individual counseling included lessons on lifestyle patterns, body composition, nutrition, physical activity, stress-related overweight or obesity, and overall health.

After the 12 sessions, an intervention continued to maintain subjects’ lifestyle modifications. Researchers monitored and encouraged subjects via telephone call or short message system every week. For emotional support, the professionals used listening, praise, and encouragement techniques throughout the entire program (Table 1). The experimental group participated in a twelve-session program; the control group was provided a booklet on health management for obese women, and after the intervention, if the subjects in the control group want to program information, LSI was summarized and provided.

### 2.5. Outcome Measures

#### 2.5.1. Health-Promoting Lifestyle Profile II (HPLP II)

Health-promoting lifestyle behaviors were measured using the Health-Promoting

Lifestyle Profile II questionnaire (HPLP-II). The HPLP was first developed by Walker et al. [22]; the HPLP II questionnaire is the revised version. This questionnaire includes six subscales (health responsibility, physical activity, nutrition, spiritual growth, interpersonal relationships, and stress management) and 52 items rated on a four-point Likert scale (ranging 1–4). The total score is the mean of responses for each subscale and for all items. Higher scores indicate better health-promoting lifestyle behavior. The Korean version of the HPLP-II has well-established validity and reliability [23]. In this study, the Cronbach’s alpha for HPLP-II was 0.92, and Cronbach’s alphas of the subscales were 0.80 for health responsibility, 0.80 for physical activity, 0.74 for nutrition, 0.90 for spiritual growth, 0.84 for interpersonal relationships, and 0.74 for stress management.

#### 2.5.2. Depression, Anxiety, and Stress Scale-21 (DASS-21)

Psychological distress was measured using the depression, anxiety, and stress scale

(DASS-21) [24]. This 21-item scale contains three subscales: depression (seven items), anxiety (seven items), and stress (seven items). Each item is scored on a four-point Likert scale ranging from 0 to 3 points; higher scores indicate higher psychological distress. The Korean version of the DASS-21 has well-established reliability and validity [25]. The Cronbach’s alpha in the current study was 0.85 for the total score, 0.73 for depression, 0.76 for anxiety, and 0.80 for stress.

#### 2.5.3. Reproductive Health

Reproductive health referred to the knowledge, attitudes, and behaviors related to reproductive health, dysmenorrhea, gynecologic symptoms, and LH/FSH ratio. (i) The reproductive health knowledge scale (35 items) was used; it measures knowledge of structure and function of the reproductive system, pregnancy, birth, contraception, sexually transmitted disease, and cancer of the reproductive system [26]. Cronbach’s alpha was 0.71 in the current study. (ii) The 12-item reproductive health attitude scale is scored on a five-point Likert scale; higher scores indicate better reproductive attitude [26]. The Cronbach’s alpha was 0.79 in the current study. (iii) The reproductive health behavior scale contains 18 items, including safe sex, sexual responsibility, genital health management, sexually transmitted disease prevention, and genital hygiene [27]. Reproductive health knowledge and attitude scales have established validity and reliability in Korean university students [13]. (iv) Dysmenorrhea was measured on a scale from no pain at all (0 points) to unbearable severe pain (10 points). (v) We identified the gynecologic symptoms among the reproductive health indicators presented by the World Health Organization (WHO) [28]: abnormal vaginal discharge, genital itching, genital pain, irregular vaginal spotting, and genital warts that occur mostly in reproductive-age women. Multiple responses were made for subjective and objective symptoms, and one or more cases were defined as having gynecologic symptoms. (vi) luteinizing hormone (LH) and follicular-stimulating hormone (FSH) were measured before the first seven days of the menstruation cycle; insufficient FSH levels and increased LH levels contribute to impaired follicular development and ovarian androgen production [29].

#### 2.5.4. Body Composition

Body composition was assessed according to body weight, BMI, body fat, abdominal fat, high-density lipoprotein (HDL),(low-density lipoprotein (LDL), triglyceride (TG), and glucose. A bioelectrical impedance analysis device (InBody 270) (InBody, Seoul, Korea) was used to examine body composition after fasting. The accuracy of this device had 93–96% in measurement of muscle, fat, and body water [30]. Blood tests were performed after fasting from midnight before the test. We identified demographic factors such as age and religion, as well as lifestyle-related characteristics such as smoking, alcohol consumption, and hours of sleep.

### 2.6. Ethical Considerations and Statistical Analysis

The study was approved by the Institutional Review Board (IRB-2017-03-006-003) at the Jeonbuk National University and the Helsinki Declaration of 1975. All subjects provided written consent after being informed of the study process, benefits, risks, and voluntary participation and withdrawal.

The SPSS version 25.0 (IBM SPSS Statistics for Windows, IBM Corp., Armonk, NY, USA) was used for statistical analyses. An independent *t*-test and chi-squared test were used to verify homogeneity between the experimental and control groups, and the normality of the data was confirmed using the Shapiro–Wilk test. Descriptive statistics were calculated to identify participants’ general characteristics. An independent t-test was used to examine differences between the pretest and posttest results to compare the intervention effects between the experimental and control groups. An analysis of covariance (ANCOVA) was used for variables that were not homogenous in the pretest; the pretest value was used as a covariate in the posttest, and the posttest value was determined. A two-tailed *p* < 0.05 value was set as statistically significant.

## 3. Results

### 3.1. Homogeneity Test for General Characteristics and Variables between Groups

No statistically significant differences were found in the pretest, with the exception of the variables for physical activity, nutrition, and LH/FSH ratio (Table 2).

### 3.2. Effects of the Lifestyle Intervention Based on the Interaction Model of Client Health Behavior

The overall HPLP-II scores in the experimental group were significantly higher than the control group (effect size (ES, Cohen’s *d*) = 0.70), and some sub-dimensions were significantly higher (ES = 0.57~0.70). Overall, DASS-21 scores were significantly lower in the experimental group than in the control group (ES = 0.97) and the sub-dimensions between groups (ES = 0.75~0.86). Scores for reproductive knowledge (ES = 0.96), attitude (ES = 0.82), and behavior (ES = 0.66) were significantly higher in the experimental group than in the control group. Finally, body weight (ES = 1.23), BMI (ES = 1.28), body fat (ES = 0.72), and TG level (ES = 0.66) significantly decreased in the experimental group compared to the control group (Table 3).

## 4. Discussion

The purpose of this study was to investigate the effects of a 12-week LSI on health-promoting-behavior, psychological distress, reproductive health, and body composition index of overweight or obese female college students based on the IMCHB. The LSI was found to be effective in improving health-promoting-behaviors, psychological distress, reproductive health, and body composition index.

LSIs are the first choice for those who are overweight or obese according to international guidelines [31]. Accordingly, it was confirmed that it is effective for various health indicators such as increasing physical exercise, improving nutritional status, and improving body composition index by applying it to various overweight and obese populations (e.g., children, college students, adults, and women) [17,18,19,21]. However, there are not many LSIs for comprehensive health-promotion of overweight and obese female college students. This study confirmed that this type of intervention could be applied to address various physical, psychological, and reproductive health problems of overweight and obese female students.

After providing the LSI in this study, the health-promotion behavior of the experimental group improved in comparison with the control group. This is consistent with previous research that provided a health-promotion intervention to 73 college students and found that among the subdomain of health-promotion behavior, nutrition was the most improved (ES = 0.70) [32]. According to the 2018 national health and nutrition survey in Korea, the rate of skipping breakfast in women in their 20 s was 54.4%, the highest among all age groups, and the energy/fat excess intake rate was also high at 6.5% [33]. Given that the LSI improved the nutritional status of female college students, it seems necessary to continuously implement LSI programs for overweight and obese female college students.

After our LSI, the experimental group showed less psychological distress than the control group. This is consistent with a systematic review and meta-analysis on LSIs provided to overweight or obese women of reproductive age, with depression declining by 1.35 times and anxiety by 1.74 times [21]. It was reported that overweight, obese women were at a higher risk of depression (1.17–1.55 times) than normal-weight women, and there was a correlation between waist circumference and anxiety [31,34]. In addition, female college students experience low body image, stigma, and low academic achievement, as well as stress due to adaptation to new environments and academic and career preparations [5]. Female college students have also been reported to experience more mental symptoms than their male counterparts [35]. This study confirmed that LSIs have a positive effect on overweight or obese female college students experiencing such psychological distress.

Further, weight loss is effective in reducing psychological distress, including depression and anxiety [21]. In this study, it can be seen that the experimental group has a weight loss of 1.69 kg, and the BMI is 0.65 kg/m^2^ compared to the control group, consistent with a previous study. However, simple diet-induced weight loss was not effective in reducing psychological distress in overweight or obese adults; instead, it was effective in reducing stress when there was continuous interaction, monitoring, and support with the subjects [36,37]. With this in mind, our LSI program employed consistent interactions between the health professionals and subjects based on the IMCHB. Participants completed an activity log that included diet, physical activity, and stress management, and the health experts conducted health counseling and monitoring on diet, physical activity, and stress management. Through this process, not only did psychological stress decrease, but participants were able to maintain a healthy lifestyle.

Moreover, after the LSI, the experimental group showed improvements in reproductive health knowledge, attitudes, and behavior scores compared to the control group. In a study of 275 female college students, healthy lifestyles were associated with high scores in reproductive health knowledge, attitudes, and behaviors [14]. It has been reported that vaginal health is related to good nutrition, physical activity, stress management, and mental health. As such, this study confirmed that reproductive health knowledge, attitudes, and behaviors are improved by adopting a healthier lifestyle.

However, there was no difference in dysmenorrhea, gyn symptoms, and LH/FSH ratio between the groups. This is contrary to previous findings that reproductive health improved after providing 8-week LSIs for middle-aged women [38]. This may be due to the age difference between the study samples. In particular, overweight and obese women in their 20 s are likely to develop polycystic ovary syndrome (PCOS) with insulin resistance and hyperinsulinemia, thus confirming a change in the LH/FSH ratio [39]. However, there are reports of subjects with PCOS with different LH/FSH ratios, and unusually higher levels of FSH than LH were reported in about 30% of cases [40]. There was no difference in LH/FSH ratio in our study; therefore, in the future, it may be necessary to confirm the ovarian functional status through a more objective test to confirm reproductive health.

In this study, after the LSI, the body weight, BMI, body fat (%), and TG were lower in the experimental group than in the control group. This is consistent with international research guidelines that positive lifestyle changes are effective as a weight-loss strategy for overweight or obese women [21,31]. It was confirmed that an LSI program that addressed healthy nutrition, improving physical activity, and stress management had a positive effect on weight loss and body composition. However, there was no difference in HDL and LDL other than TG. This shows that weight loss in the experimental group was 1.69 kg; thus, the degree of weight loss was small enough to show hematological changes. It will be necessary to confirm various hematologic indicators through the application of a long-term program in the future.

Since the LSI program contributed to the health-promotion of female students, it is necessary to provide for students by reflecting the LSI program in the university’s school life program or welfare promotion program. Furthermore, it is necessary to conduct research that applies to the broader population of obese women in the future.

There are some limitations to this study. First, since the study sample was from one specific region and quasi-experimental control study, the findings might not generalize to the entire overweight or obese female population; also, there would be a selection bias. We propose a large-scale randomized control study to confirm the results by minimizing the selection bias. Second, this study lasted 12 weeks; however, there were limitations in identifying the effects of biochemical indicators, and since it is necessary to maintain the normal weight, long-term studies are needed. In addition, only the subjective symptom report and LH/FSH ratio were confirmed as indicators of reproductive health, but it is necessary to monitor the health status of the ovaries and uterus through more objective tests such as CT scans or ultrasound. Nevertheless, this study identified that an LSI has positive effects on health-promoting lifestyle behaviors, psychological distress, reproductive health, and body composition, including body weight, BMI, body fat and TG level in overweight and obese female college students.

## 5. Conclusions

In sum, it was confirmed that LSIs have a significant effect on improving health-promoting-behaviors in overweight or obese female college students, reducing psychological distress, and improving reproductive health, weight loss, and body composition. Therefore, our findings are significant since they confirm that LSIs centered on the interactions between the subject and health experts are effective for overweight or obese female college students. Accordingly, it is expected that the health of women in college, which is critical for their future health, will be promoted via LSIs.

## Figures and Tables

**Table 1:** Contents of the lifestyle intervention.

Session	Contents	Interaction	Method	Minutes
1	Introduction the program contents and schedule	Individual		
	Health information: the importance of a healthy lifestyle for physical, psychological and reproductive health Provision of an educational booklet including a diary for food and daily activity		Face-to-face	60
	Affective support: encouragement and reinforcement for participating in the program			
2	Health information: nutrition for weight control (low-calorie diet, healthy diet) Lifestyle monitoring Affective support: encouragement and reinforcement of healthy lifestyle	IndividualGroup	Face-to-face	60
3	Individual health counseling: lifestyle patterns and body composition Lifestyle monitoring Affective support: encouragement and reinforcement of healthy lifestyle	Individual	Face-to-face	60
4	Health information: the importance of physical activity Physical activity: flexibility training, resistance training, neurovascular training Lifestyle monitoring	IndividualGroup	Face-to-face	60
	Affective support: encouragement and reinforcement of healthy lifestyle			
5	Individual health counseling: physical activity Lifestyle monitoring Affective support: encouragement and reinforcement of healthy lifestyle	Individual	Face-to-face	60
6	Health information: stress management (knowing oneself) Lifestyle monitoring Affective support: encouragement and reinforcement of healthy lifestyle	IndividualGroup	Face-to-face	60
7	Individual health counseling: stress-related overweight or obesity Lifestyle monitoring Affective support: encouragement and reinforcement of healthy lifestyle	Individual	Face-to-face	60
8	Health information: nutrition for weight control (diet behaviors and recipes) Lifestyle monitoring Affective support: encouragement and reinforcement of healthy lifestyle	IndividualGroup	Face-to-face	60
9	Individual health counseling: overall health Lifestyle monitoring Affective support: encouragement and reinforcement of healthy lifestyle	Individual	Face-to-face	60
10	Health information: various methods of physical exercise Physical activity: flexibility training, resistance training, neurovascular training Affective support: encouragement and reinforcement of healthy lifestyle	IndividualGroup	Face-to-face	60
11	Health information: stress management (strategies for self-respecting) Lifestyle monitoring Affective support: encouragement and reinforcement of healthy lifestyle	IndividualGroup	Face-to-face	60
12	Lifestyle monitoring: support, reinforcement, and reward Evaluation of the program	Individual	Face-to-face	60

**Table 2:** Homogeneity test for participant characteristics and variables in the experimental and control groups (N = 44).

Characteristics	Classification	Exp (n = 21)	Cont (n = 23)	χ^2^, t or z	*p*
		M ± SD or n (%)	M ± SD or n (%)		
General Characteristics					
Age (years)		22.7 ± 2.5	22.1 ± 2.6	0.805	0.425
Religion	Yes	8 (38.1)	8 (34.8)	0.052	1.000
	No	13 (61.9)	15 (65.2)		
Smoking	Yes	2 (9.5)	1 (4.3)	0.463	0.599
	No	19 (90.5)	22 (95.7)		
Alcohol consumption	Yes	4 (19.0)	7 (30.4)	0.759	0.494
	No	17 (81.0)	16 (69.6)		
Hours of sleep		6.55 ± 1.45	6.52 ± 1.51	0.058	0.954
Variables					
*HPLP II*		2.42 ± 0.30	2.24 ± 0.39	1.684	0.100
HR		2.13 ± 0.48	1.88 ± 0.49	1.684	0.100
PA		2.08 ± 0.52	1.75 ± 0.49	2.182	0.035
NUT		2.25 ± 0.43	1.90 ± 0.52	2.436	0.019
SG		2.60 ± 0.40	2.52 ± 0.58	0.502	0.618
IR		3.02 ± 047	3.16 ± 0.63	−0.903	0.372
SM		2.42 ± 0.43	2.20 ± 0.53	1.545	0.130
*DASS-21*		15.71 ± 6.96	15.09 ± 0.30	0.252	0.803
Depression		4.19 ± 3.34	4.52 ± 3.02	−0.346	0.731
Anxiety		3.62 ± 3.11	3.52 ± 3.41	0.314	0.922
Stress		7.91 ± 3.40	5.04 ± 4.82	0.679	0.501
*Reproductive health*					
Knowledge		21.24 ± 4.38	22.48 ± 3.40	−0.986	0.330
Attitude		46.86 ± 5.24	48.61 ± 5.30	−1.101	0.277
Behavior		62.71 ± 3.49	61.83 ± 4.58	0.718	0.477
Dysmenorrhea		3.93 ± 2.51	4.50 ± 2.54	−0.749	0.458
Gynecologic symptom (n)		0.71 ± 0.78	0.52 ± 0.90	0.755	0.455
Yes		11 (52.4)	7 (30.4)	2.187	0.220
No		10 (47.6)	16 (69.6)		
LH/FSH ratio		0.67 ± 0.42	1.11 ± 0.52	−3.082	0.004
*Body composition*					
BW (kg)		68.65 ± 10.04	67.68 ± 9.68	0.326	0.746
BMI (kg/m^2^)		26.33 ± 2.68	26.05 ± 3.57	0.295	0.770
Body fat (%)		36.63 ± 4.40	35.93 ± 6.17	0.428	0.671
Abdominal fat (%)		0.86 ± 0.05	0.88 ± 0.04	−1.691	0.098
HDL		56.38 ± 10.91	57.09 ± 14.10	−0.184	0.855
LDL		105.90 ± 30.87	107.00 ± 24.72	−0.130	0.897
TG		89.43 ± 38.42	91.48 ± 26.62	−0.207	0.837
Glucose		84.71 ± 8.64	84.09 ± 8.01	0.250	0.804

Cont: control group; Exp: experimental group; HPLP II: health-promoting lifestyle profile II; HR: health responsibility; PA: physical activity; NUT: nutrition; SG: spiritual growth; IR: interpersonal relationships; SM: stress management; DASS-21: depression anxiety stress scale-21; LH: luteinizing hormone; FSH: follicular-stimulating hormone; BW: body weight; BMI: body mass index; HDL: high-density lipoprotein; LDL: low-density lipoprotein; TG: triglyceride.

**Table 3:** Effects of the lifestyle intervention on health-promoting lifestyle, psychological distress, reproductive health, and body composition in the experimental and control groups (N = 44).

Characteristics	Groups	Pretest (before LSI)	Posttest (after 12-Week LSI)	Difference (Post-Pre)	t, F, χ^2^, or z	*p*	*Cohen’s* *d*
		M ± SD or n (%)	M ± SD or n (%)	M ± SD or n (%)			
*HPLP II*	Exp	2.42 ± 0.30	2.65 ± 0.45	0.23 ± 0.32	2.311	0.026	0.70
	Cont	2.24 ± 0.39	2.27 ± 0.42	0.02 ± 0.28			
HR	Exp	2.13 ± 0.48	2.26 ± 0.57	0.13 ± 0.42	0.937	0.354	0.28
	Cont	1.88 ± 0.49	1.90 ± 0.47	0.02 ± 0.38			
PA ^a^	Exp	2.08 ± 0.52	2.48 ± 0.68	0.40 ± 0.62	7.718	0.011	0.57
	Cont	1.75 ± 0.49	1.80 ± −0.60	0.05 ± 0.61			
NUT ^a^	Exp	2.25 ± 0.43	2.81 ± 0.96	0.56 ± 0.78	4.531	0.039	0.70
	Cont	1.90 ± 0.52	2.02 ± 0.58	0.13 ± 0.41			
SG	Exp	2.60 ± 0.40	2.52 ± 0.62	−0.08 ± 0.58	−0.069	0.946	0.14
	Cont	2.52 ± 0.58	2.53 ± 0.67	0.00 ± 0.53			
IR	Exp	3.02 ± 047	3.12 ± 0.49	0.10 ± 0.44	2.116	0.040	0.64
	Cont	3.16 ± 0.63	3.00 ± 0.53	−0.16 ± 0.37			
SM	Exp	2.42 ± 0.43	2.71 ± 0.60	0.29 ± 0.44	1.516	0.137	0.45
	Cot	2.20 ± 0.53	2.29 ± 0.56	0.10 ± 0.40			
*DASS-21*	Exp	15.71 ± 6.96	11.29 ± 8.91	−5.48 ± 8.00	−3.197	0.003	0.97
	Cont	15.09 ± 0.30	16.26 ± 12.81	3.57 ± 10.47			
Depression	Exp	4.19 ± 3.34	2.95 ± 3.50	−1.52 ± 2.80	−2.504	0.016	0.75
	Cont	4.52 ± 3.02	4.78 ± 4.75	1.13 ± 4.05			
Anxiety	Exp	3.62 ± 3.11	2.33 ± 1.91	−1.29 ± 2.78	−2.841	0.007	0.86
	Cont	3.52 ± 3.41	4.48 ± 3.82	1.30 ± 3.23			
Stress	Exp	7.91 ± 3.40	6.00 ± 4.46	−2.67 ± 4.78	−2.638	0.012	0.80
	Cont	5.04 ± 4.82	7.00 ± 5.54	1.13 ± 4.76			
*Reproductive health*							
Knowledge	Exp	21.24 ± 4.38	23.52 ± 3.64	2.77 ± 3.74	3.180	0.003	0.96
	Cont	22.48 ± 3.40	23.04 ± 4.48	−0.39 ± 2.81			
Attitude	Exp	46.86 ± 5.24	48.95 ± 6.00	2.96 ± 3.29	2.731	0.009	0.82
	Cont	48.61 ± 5.30	47.04 ± 9.55	−2.17 ± 8.00			
Behavior	Exp	62.71 ± 3.49	65.24 ± 5.39	3.67 ± 4.69	2.167	0.036	0.66
	Cont	61.83 ± 4.58	62.17 ± 7.20	0.26 ± 5.63			
Dysmenorrhea	Exp	3.93 ± 2.51	3.43 ± 2.51	−0.50 ± 1.26	−1.322	0.193	0.40
	Cont	4.50 ± 2.54	4.48 ± 2.43	0.15 ± 1.91			
Gyn symptom (n)	Exp	0.71 ± 0.78	0.48 ± 0.60	−0.24 ± 0.77	−0.091	0.928	0.03
	Cont	0.52 ± 0.90	0.30 ± 0.47	−0.22 ± 0.74			
Yes	Exp	11 (52.4)	9 (42.9)		0.732	0.533	
	Cont	7 (30.4)	7 (30.4)				
LH/FSH ratio ^a^	Exp	0.67 ± 0.42	0.96 ± 0.64	0.26 ± 0.54	0.032	0.586	0.53
	Con	1.11 ± 0.52	1.05 ± 0.52	−0.06 ± 0.66			
*Body composition*							
BW (kg)	Exp	68.65 ± 10.04	66.97 ± 9.30	−1.69 ± 2.43	−4.087	<0.001	1.23
	Cont	67.68 ± 9.68	68.49 ± 9.62	0.81 ± 1.57			
BMI (kg/m^2^)	Exp	26.33 ± 2.68	25.68 ± 2.46	−0.65 ± 0.89	−4.235	<0.001	1.28
	Cont	26.05 ± 3.57	26.38 ± 3.70	0.33 ± 0.63			
Body fat (%)	Exp	36.63 ± 4.40	36.37 ± 4.94	−0.26 ± 2.05	−2.402	0.021	0.72
	Cont	35.93 ± 6.17	37.70 ± 5.13	1.76 ± 3.32			
Abdominal fat (%)	Exp	0.86 ± 0.05	0.88 ± 0.04	0.02 ± 0.03	−0.657	0.515	0.28
	Cont	0.88 ± 0.04	0.91 ± 0.05	0.03 ± 0.04			
HDL	Exp	56.38 ± 10.91	59.19 ± 11.52	2.81 ± 8.23	0.588	0.559	0.18
	Cont	57.09 ± 14.10	58.22 ± 11.06	1.13 ± 10.45			
LDL	Exp	105.90 ± 30.87	98.38 ± 27.38	−7.53 ± 13.73	−0.074	0.941	0.02
	Cont	107.00 ± 24.72	99.87 ± 17.35	−7.13 ± 20.33			
TG	Exp	89.43 ± 38.42	79.67 ± 27.75	−9.76 ± 33.50	−2.188	0.034	0.66
	Cont	91.48 ± 26.62	101.00 ± 31.34	9.52 ± 24.66			
Glucose	Exp	84.71 ± 8.64	83.05 ± 8.68	−1.67 ± 8.82	−0.344	0.732	0.10
	Cont	84.09 ± 8.01	83.48 ± 9.77	−0.61 ± 11.28			

Cont: control group; Exp: experimental group; Gyn: gynecologic; HPLP II: health-promoting lifestyle profile II; HR: health responsibility; PA: physical activity; NUT: nutrition; SG: spiritual growth; IR: interpersonal relationships; SM: stress management; DASS-21: depression anxiety stress scale-21; LH: luteinizing hormone; FSH: follicular-stimulating hormone; BW: body weight; BMI: body mass index; HDL: high-density lipoprotein; LDL: low-density lipoprotein; TG: triglyceride. ^An^ ANCOVA.

## Data Availability

The data presented in this study are available on request from the corresponding author. The data are not publicly available due to privacy.

## References

[1] Hales C.M., Carroll M.D., Fryar C.D., Ogden C.L. (2017). Prevalence of Obesity among Adults and Youth: United States, 2015–2016. NCHS Data Brief.

[2] Korean Statistical Information Service Obesity Prevalence Trend. http://kosis.kr/statHtml/statHtml.do?orgId=117&tblId=DT_11702_N101&vw_cd=MT_ZTITLE&list_id=117_11702_B01&seqNo=&lang_mode=ko&language=kor&obj_var_id=&itm_id=&conn_path=MT_ZTITLE.

[3] Egger G., Binns A., Rössner S., Sagner M. (2017). Lifestyle Medicine: Lifestyle, the Environment, and Preventive Medicine in Health and Disease.

[4] Zhang Y.-X., Wang S.-R., Zhao J.-S., Chu Z.-H. (2016). Prevalence of overweight and central obesity and their relationship with blood pressure among college students in Shandong, China. Blood Press. Monit..

[5] Desai M.N., Miller W.C., Staples B., Bravender T. (2008). Risk Factors Associated with Overweight and Obesity in College Students. J. Am. Coll. Health.

[6] Odlaug B.L., Lust K., Wimmelmann C.L., Chamberlain S.R., Mortensen E.L., Derbyshire K., Christenson G., Grant J.E. (2015). Prevalence and correlates of being overweight or obese in college. Psychiatry Res..

[7] Chu S.Y., Kim S.Y., Lau J., Schmid C.H., Dietz P.M., Callaghan W.M., Curtis K.M. (2007). Maternal obesity and risk of stillbirth: A metaanalysis. Am. J. Obstet. Gynecol..

[8] Maheshwari A., Stofberg L., Bhattacharya S. (2007). Effect of overweight and obesity on assisted reproductive technology—A systematic review. Hum. Reprod. Updat..

[9] Greaney M.L., Less F.D., White A.A., Dayton S.F., Riebe D., Blissmer B., Shoff S., Walsh J.R., Greene G.W. (2009). College Students’ Barriers and Enablers for Healthful Weight Management: A Qualitative Study. J. Nutr. Educ. Behav..

[10] Jakubiec D., Kornafel D., Cygan A., Górska-Kłęk L., Chromik K. (2015). Lifestyle of students from different universities in Wroclaw, Poland. Roczniki Państwowego Zakładu Higieny.

[11] Yahia N., Wang D., Rapley M., Dey R. (2016). Assessment of weight status, dietary habits and beliefs, physical activity, and nutritional knowledge among university students. Perspect. Public Health.

[12] Ahn S.-H., Um Y.-J., Kim Y.-J., Kim H.-J., Oh S.-W., Lee C.M., Kwon H., Joh H.-K. (2016). Association between Physical Activity Levels and Physical Symptoms or Illness among University Students in Korea. Korean J. Fam. Med..

[13] Nho J.-H., Kim H.S. (2019). Gender Differences and Relationships among Lifestyle and Reproductive Health in University Students. Korean J. Women Health Nurs..

[14] Nho J.-H., Yoo S.-H. (2018). Relationships among Lifestyle, Depression, Anxiety, and Reproductive Health in Female University Students. Korean J. Women Health Nurs..

[15] Bavil D.A., Dolatian M., Mahmoodi Z., Baghban A.A. (2016). Comparison of lifestyles of young women with and without primary dysmenorrhea. Electron. Physician.

[16] Sabharwal A.M. (2014). Effectiveness of Lifestyle Interventions among College Students: An Overview. J. Nutr. Food Sci..

[17] Dour C.A., Horacek T.M., Schembre S.M., Lohse B., Hoerr S., Kattelmann K., White A.A., Shoff S., Phillips B., Greene G. (2013). Process Evaluation of Project WebHealth: A Nondieting Web-based Intervention for Obesity Prevention in College Students. J. Nutr. Educ. Behav..

[18] Shahril M.R., Dali W.P.E.W., Lua P.L. (2013). A 10-Week Multimodal Nutrition Education Intervention Improves Dietary Intake among University Students: Cluster Randomised Controlled Trial. J. Nutr. Metab..

[19] Baillot A., Romain A.J., Boisvert-Vigneault K., Audet M., Baillargeon J.P., Dionne I.J., Valiquette L., Chakra C.N.A., Avignon A., Langlois M.-F. (2015). Effects of Lifestyle Interventions That Include a Physical Activity Component in Class II and III Obese Individuals: A Systematic Review and Meta-Analysis. PLoS ONE.

[20] Cox C.L. (1982). An interaction model of client health behavior: Theoretical prescription for nursing. Adv. Nurs. Sci..

[21] Van Dammen L., Wekker V., De Rooij S.R., Groen H., Hoek A., Roseboom T.J. (2018). A systematic review and meta-analysis of lifestyle interventions in women of reproductive age with overweight or obesity: The effects on symptoms of depression and anxiety. Obes. Rev..

[22] Walker S.N., Sechrist K.R., Pender N.J. (1987). The Health-Promoting Lifestyle Profile: Development and psychometric characteristics. Nurs. Res..

[23] Noh J.-W., Yun H.-Y., Park H., Yu S.-E. (2015). A Study of Predictive Factors Affecting Health: Promoting Behaviors of North Korean Adolescent Refugees. J. Prev. Med. Public Health.

[24] Henry J.D., Crawford J.R. (2005). The short-form version of the Depression Anxiety Stress Scales (DASS-21): Construct validity and normative data in a large non-clinical sample. Br. J. Clin. Psychol..

[25] Cha E.S. Cha Korean Translation of the DASS21. http://www2.psy.unsw.edu.au/Groups/Dass/Korean/Korean%20Cha.htm.

[26] Park M.N., Choi S.Y. (2014). Development of Reproductive Health Program and Identification of Effect for Married Women Immigrants. J. Korean Acad. Nurs..

[27] Jo H.Y., Kim Y.H., Son H.M. (2014). Development of a Scale to Measure Reproductive Health Promoting Behavior of Undergraduates. Korean J. Health Educ. Promot..

[28] World Health Organization Sexual and Reproductive Health. https://www.who.int/reproductivehealth/en/.

[29] Blank S., McCartney C., Marshall J. (2006). The origins and sequelae of abnormal neuroendocrine function in polycystic ovary syndrome. Hum. Reprod. Updat..

[30] Ling C.H., de Craen A.J., Slagboom P.E., Gunn D.A., Stokkel M.P., Westendorp R.G., Maier A.B. (2011). Accuracy of direct segmental multi-frequency bioimpedance analysis in the assessment of total body and segmental body composition in middle-aged adult population. Clin. Nutr. (Edinb. Scotl.).

[31] Kim M.K., Lee W.-Y., Kang J.-H., Kang J.-H., Kim B.T., Kim S.M., Kim E.M., Suh S.-H., Shin H.J., Lee K.R. (2014). 2014 Clinical Practice Guidelines for Overweight and Obesity in Korea. Endocrinol. Metab..

[32] Díez S.M.U., Fortis A.P., Franco S.F. (2012). Efficacy of a Health-Promotion Intervention for College Students: A Randomized Controlled Trial. Nurs. Res..

[33] Korea Centers for Disease Control and Prevention 2018 Korea Health Statistics Korea. https://knhanes.cdc.go.kr/knhanes/sub04/sub04_03.do?classType=7.

[34] Luppino F.S., De Wit L.M., Bouvy P.F., Stijnen T., Cuijpers P., Penninx B.W.J.H., Zitman F.G. (2010). Overweight, Obesity, and Depression: A Systematic Review and Meta-Analysis of Longitudinal Studies. Arch. Gen. Psychiatry.

[35] De Wit L.M., Fokkema M., Van Straten A., Lamers F., Cuijpers P., Penninx B.W.J.H. (2010). Depressive and anxiety disorders and the association with obesity, physical, and social activities. Depress. Anxiety.

[36] Booth A.O., Wang X., Turner A.I., Nowson C.A., Torres S.J. (2018). Diet-Induced Weight Loss Has No Effect on Psychological Stress in Overweight and Obese Adults: A Meta-Analysis of Randomized Controlled Trials. Nutrients.

[37] Imayama I., Alfano C.M., Kong A., E Foster-Schubert K., E Bain C., Xiao L., Duggan C., Wang C.-Y., Campbell K.L., Blackburn G.L. (2011). Dietary weight loss and exercise interventions effects on quality of life in overweight/obese postmenopausal women: A randomized controlled trial. Int. J. Behav. Nutr. Phys. Act..

[38] Nho J.-H., Hwang E.S. (2019). Effects of Multidisciplinary Lifestyle Modification Program on Health-promoting Behavior, Psychological Distress, Body Composition and Reproductive Symptoms among Overweight and Obese Middle-aged Women. Korean J. Adult Nurs..

[39] DeUgarte C.M., A Bartolucci A., Azziz R. (2005). Prevalence of insulin resistance in the polycystic ovary syndrome using the homeostasis model assessment. Fertil. Steril..

[40] Malini N., George K.R. (2018). Evaluation of different ranges of LH:FSH ratios in polycystic ovarian syndrome (PCOS)—Clinical based case control study. Gen. Comp. Endocrinol..

